# Surgical Hand Scrubbing: A Clinical Audit at a Referral Hospital

**DOI:** 10.7759/cureus.96465

**Published:** 2025-11-10

**Authors:** Elshazalia Ahmed Omer Ahmed, Zomoroda Hassen Saeed Mohamed, Malaz S Sayed, ELafraa Ahmes Mohamed Ahmed, Afraa Merghani Abd Alazim Bala, Mujtaba A Abdelrahem, Ahmed Galal Abdelmaged Mohamedahmed

**Affiliations:** 1 General Surgery, University of Khartoum, Khartoum, SDN; 2 General Surgery, Royal College of Surgeons of England, London, GBR; 3 General Surgery, Ibrahim Malik Teaching Hospital, Khartoum, SDN; 4 Surgery, Royal College of Surgeons in Ireland, Dublin, GBR; 5 Medicine, University of Bahri, Khartoum, SDN; 6 General Surgery, Wad Madani Teaching Hospital, Wad Madani, SDN; 7 Anatomical Sciences, National University, Khartoum, SDN; 8 General Practice, University of Khartoum, Khartoum, SDN; 9 General Surgery, Royal College of Surgeons of Edinburgh, Edinburgh, GBR; 10 General Practice, Ibrahim Malik Teaching Hospital, Khartoum, SDN; 11 General Surgery, Prince Osman Digna Referral Hospital, Khartoum, SDN; 12 Medicine, University of Khartoum, Khartoum, SDN; 13 Medical Education, Al Neelain University, Khartoum, SDN; 14 Surgery, Red Sea University, Port Sudan, SDN; 15 Surgery, Royal College of Physicians and Surgeons of Glasgow, Glasgow, GBR; 16 Medicine, University of Sinnar, Sinnar, SDN; 17 Surgery, Royal College of Surgeons of Edinburgh, Edinburgh, GBR

**Keywords:** audit cycle, compliance, hand hygiene, infection control, surgical site infections, who guidelines

## Abstract

Background

Surgical site infections (SSIs) continue to be a serious challenge in the medical profession, particularly in developing countries, where they are responsible for a notable percentage of hospital-acquired infections. Although proper hand hygiene continues to be a mainstay for the prevention of SSIs, compliance by surgical teams tends to be less than optimal. Factors such as limited time, inadequate training, and resource constraints are among the reasons behind this persisting problem. This study critically examines and seeks to improve compliance with surgical hand scrubbing protocols at Prince Osman Digna Referral Hospital and referencing standards established by the World Health Organization (WHO).

Methods

The study employed a three-cycle observational cross-sectional audit conducted from October to December 2024, with each cycle comprising 30 recorded observations. The first cycle established baseline compliance. Following this, targeted interventions, such as instructional videos, practical training sessions, and direct feedback, were introduced. In the next cycle, adherence is re-evaluated. Then, in the final cycle, the same interventions were repeated and reinforced through in-person live demonstrations. Data collection relied on the use of a systematic checklist, allowing for the quantitative analysis of compliance rates and the qualitative identification of barriers to adherence.

Results

Following the introduction of training and feedback, compliance with hand hygiene protocols increased from 82% to 96% between the last two cycles. The most significant improvement was seen within a minimum time of two minutes during scrubbing, rising by 166%. On the other sites, for instance, drying hands and forearms with a sterile towel also improved significantly, from 30% to 42.8%. Overall, these findings highlight the incredible impact that organized education and ongoing feedback can have on following proper hand hygiene protocols.

Conclusion

Interventions targeted at education resulted in notable gains in adherence to surgical hand scrubbing protocols, thereby enhancing infection control measures. These findings highlight the effectiveness of structured training. That said, relying on one-off initiatives isn't enough. Sustained educational efforts and long-term strategies are needed to maintain compliance and achieve further reductions in SSIs.

## Introduction

Surgical site infections (SSIs) emerge from the interplay of risk factors associated with the patient, surgeon, and healthcare environment. Microorganisms that cause SSIs originate from multiple sources within the operating room, including the hands of the surgical team. Surgical hand preparation (SHP) has historically been essential in preventing SSIs [1,2]. Hand hygiene is a fundamental aspect of infection prevention and control (IPC) and is crucial in healthcare delivery across various resource settings [1]. The skin harbors two primary types of microorganisms: resident and transient or contaminant flora. While resident organisms, in general, have low pathogenic potential and are difficult to remove mechanically, transient organisms have a higher pathogenic potential [3].

Surgical hand scrubbing is a critical component of infection prevention in the operating theatre. In low-socioeconomic healthcare settings, maintaining consistent adherence to hand scrubbing protocols can be challenging due to limited resources, inadequate training, and a lack of monitoring systems. This audit was conducted to assess compliance with standard surgical hand scrubbing guidelines among operating room staff at a referral hospital and to identify gaps requiring quality improvement.

Transient microorganisms generally do not proliferate on healthcare workers' hands; however, they can persist for as long as 150 hours [4]. Studies showed that approximately 40% of healthcare workers' hands are contaminated with transient microorganisms during patient care, contributing to healthcare-associated infections, including SSIs [1]. In many developing countries, adherence to hand hygiene protocols remains worryingly low, often at just 20-30%. By contrast, high-income countries typically report compliance rates of 50-60%. Unsurprisingly, this gap contributes to SSI rates reaching as high as 30% in specific procedures.

Microorganisms are frequently acquired by healthcare workers during patient care and are responsible for the majority of healthcare-associated infections, as well as the dissemination of antimicrobial drug resistance. Consequently, the hands of healthcare workers serve as essential vectors for the transmission of microorganisms in the clinical setting, including to patients [5]. SHP differs from standard hand hygiene by focusing on the elimination of transient flora and the sustained reduction of resident flora [1] before surgical procedures.

The World Health Organization (WHO) Guidelines on Hand Hygiene in Healthcare, published in 2009, stipulate that agents used for SHP must effectively reduce microorganisms on intact skin, be non-irritating, demonstrate broad-spectrum activity, and possess both rapid action and persistence [1].

The fast-paced environment of Sudan's operating rooms, combined with constrained training periods and insufficient numbers of highly qualified medical personnel, hinders general surgery residents and house officers from achieving proficiency in hand hygiene. Adhering to the WHO Guidelines on Hand Hygiene in Healthcare is essential before any surgical procedure. The WHO highlights that promoting effective hand hygiene practices, rather than solely concentrating on the products employed, can substantially influence behavior. The objective of this audit was to assess the handwashing practices of residents and house officers in relation to SSI in the general surgery operating theatre.

The primary objective of this audit was to evaluate the adherence of surgical staff to standard hand scrubbing protocols in a referral hospital. Secondary objectives included identifying common deviations from the protocol and proposing practical recommendations to enhance compliance.

## Materials and methods

Proper surgical hand hygiene is directly linked to a reduction in SSIs, which remain a major cause of postoperative morbidity, mortality, and financial burden in low-resource hospitals. By assessing compliance and identifying barriers, this audit aims to contribute to improving infection control practices in similar healthcare settings.

Our team investigated the Department of General Surgery at Prince Osman Digna Referral Hospital in Khartoum, Sudan, a secondary hospital located in the capital of Western Sudan. Thirty residents were involved in the study in October 2024. The Quality Improvement Committee/Hospital Ethics Committee of Prince Osman Digna Referral Hospital issued approval 50/b/1.

Three cycles, each with a duration of two weeks, were conducted. Each participant provided verbal consent, and two of our team's doctors observed residents as they cleaned their hands in preparation for surgery. The WHO handwashing checklist (Table 1) was used to evaluate their performance. "Yes" was used to indicate that the candidate completed the step correctly, and "No" was used to indicate that the candidate skipped the step or did not complete it correctly. The analysis used percentages to determine the degree of compliance with each step and to evaluate how effectively each participant fulfilled all steps.

**Table 1:** WHO's handwashing checklist WHO: World Health Organization

WHO checklist [1]
1. Remove all hand accessories
2. Make sure that fingernails are clean and short
3. Wet hands and forearms with water
4. Apply soap over the whole surface of the hands and forearms
5. Rub hands palm to palm
6. Scrub the right palm over the dorsum of the left hand and vice versa
7. Rub hands palm to palm while fingers interlaced
8. The back of the fingers rubbed against the opposite palm
9. Rubbing of the clasped right thumb in the left palm in rotational movement and vice versa
10. Circular backward and forward rubbing of clasped right fingers in the left palm and vice versa
11. Rub the left wrist with the right hand and scrub up to the elbow and vice versa
12. Rinse hands and forearms under running water, allowing the water to flow from fingertips to elbow in one direction
13. Minimum time of 2 minutes during scrubbing
14. Hold hands above the elbow during rinsing and drying
15. Use a sterile towel to dry hands and forearms

Following the initial two weeks of observation, participants attended a presentation highlighting why hand hygiene matters before surgical procedures and its impact on SSI rate and featuring both a live demonstration and a video that outlined proper handwashing techniques according to WHO guidelines. Additionally, we then displayed an illustrated handwashing instruction poster in the operating room to reinforce these practices.

In the second phase, all participants underwent renewed observation to assess whether they adhered to proper handwashing practices. We re-evaluated each individual to determine their compliance with established hand scrubbing protocols. The compliance rate for each step was determined separately. The combined compliance scores were then summed to determine the overall average improvement in adherence to the hand scrubbing guidelines.

After the second cycle, results were announced, and each participant received their demonstration and was instructed to read the posters. Using the checklist, participants were observed for the third cycle. We analyzed the collected data. Then, percentages were used to reflect the average improvement in compliance.

## Results

The handwashing practices of 30 surgical participants were thoroughly observed after they were randomly assigned to one of two groups. During the initial observation, 82% (n=30) of participants followed the recommended hand hygiene procedures. In the second cycle, the compliance percentage remained unchanged at 82% (n=30), with only minor variations observed in some criteria items (Table 2). Subsequently, a third cycle was implemented. Participants were informed to read the posters that had been previously placed in the theatre. A significant increase in the success rate to 96% (n=30) demonstrated the efficacy of the interventions (Table 3). The total mean compliance rate after evaluation increased as a result of significant increases in adherence rates for each criterion, with a 17% (n=30) increase in overall compliance.

**Table 2:** Comparison of pre-evaluation and post-evaluation results: compliance percentages for various hand scrubbing steps among the first and second cycles WHO: World Health Organization

WHO checklist	Adherence rate	
	First cycle N(30)%	Second cycle N(30)%
1. Remove all hand accessories	93	93.3
2. Make sure that fingernails are clean and short	100	100
3. Wet hands and forearms with water	90	87
4. Apply soap over the whole surface of the hands and forearms	90	87
5. Rub hands palm to palm	77	74
6. Scrub the right palm over the dorsum of the left hand and vice versa	87	83.8
7. Rub hands palm to palm while fingers interlaced	90	90
8. The back of the fingers rubbed against the opposite palm	90	90
9. Rubbing of the clasped right thumb in the left palm in rotational movement and vice versa	87	86.6
10. Circular backward and forward rubbing of clasped right fingers in the left palm and vice versa	87	86.6
11. Rub the left wrist with the right hand and scrub up to the elbow and vice versa	80	80
12. Rinse hands and forearms under running water, allowing the water to flow from fingertips to elbow in one direction	87	86.6
13. Minimum time of 2 minutes during scrubbing	30	30
14. Hold hands above the elbow during rinsing and drying	73	73
15. Use a sterile towel to dry hands and forearms	70	70
Overall	82	82

**Table 3:** Comparison of pre-evaluation and post-evaluation results: enhancement in compliance percentages for various hand scrubbing steps between the second and third cycles WHO: World Health Organization

WHO checklist	Adherence rate		Improvement
	Second cycle N(30)%	Third cycle N(30)%	
1. Remove all hand accessories	93.3	100	7
2. Make sure that fingernails are clean and short	100	100	0
3. Wet hands and forearms with water	87	96.6	11
4. Apply soap over the whole surface of the hands and forearms	87	100	14.9
5. Rub hands palm to palm	74	100	35
6. Scrub the right palm over the dorsum of the left hand and vice versa	83.8	100	19.3
7. Rub hands palm to palm while fingers interlaced	90	100	11
8. The back of the fingers rubbed against the opposite palm	90	100	11
9. Rubbing of the clasped right thumb in the left palm in rotational movement and vice versa	86.6	96.6	11.5
10. Circular backward and forward rubbing of clasped right fingers in the left palm and vice versa	86.6	90	3.9
11. Rub the left wrist with the right hand and scrub up to the elbow and vice versa	80	93.3	6.7
12. Rinse hands and forearms under running water, allowing the water to flow from fingertips to elbow in one direction	86.6	90	3.4
13. Minimum time of 2 minutes during scrubbing	30	80	166
14. Hold hands above the elbow during rinsing and drying	73	93.3	27.8
15. Use a sterile towel to dry hands and forearms	70	100	42.8
Overall	82	96	17

**Figure 1:**
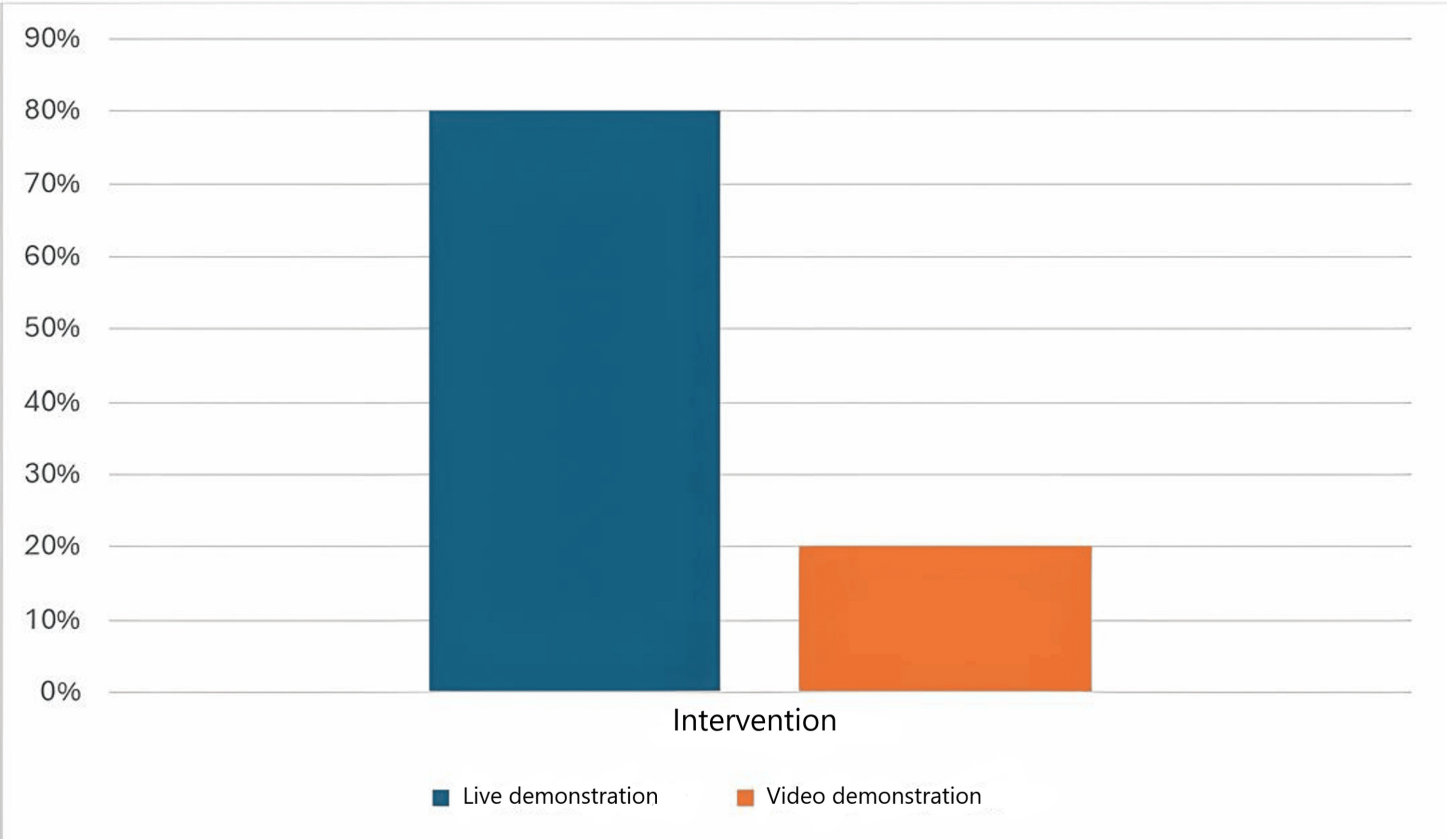
Best intervention method according to the participants

The criterion "minimum time of 2 minutes scrubbing" had the most significant improvement, from 30% to 80%, representing a 166% improvement (Table 2). This enhancement highlights how real-world examples can effectively boost participants' adherence to proper handwashing practices. To better understand the conventional hand scrubbing technique, we also asked the participants about the most successful intervention. Eighty-five percent (n=30) of the participants believed that live, hands-on demonstrations were a more effective approach to learning the technique than video presentations (Figure 1).

A 96% compliance rate was observed in this study regarding the removal of hand attachments before scrubbing. In the pre-intervention phase, ensuring that fingernails were clean and short demonstrated the highest compliance (100%). In comparison, a minimum time of two minutes during scrubbing (30%) demonstrated the lowest compliance in the handwashing and rinsing procedures, which showed significant variation in compliance across various steps. None of the participants in our audit finished the required handwashing criteria, but after receiving feedback, compliance increased to 96% after three cycles.

## Discussion

The WHO defines surgical hand scrubbing as the preparation of hands using antimicrobial soap and water before surgery [1]. Hand scrubbing was first introduced in the 19th century by Dr. Semmelweis, who observed an increase in infections among women during childbirth when health professionals handled them after contact with cadaverous materials in the dissecting room [6].

Subsequently, evidence has shown that hand contamination is a key factor in the development of SSIs, which can be controlled primarily in opposition to most other predisposing factors, such as obesity, age, or immunosuppression. Furthermore, although the invention of gloves may sound appealing, it has been observed that they are not fully protective against microbial transmission, as 18% of gloves are punctured after surgical procedures. This makes surgical scrubbing a crucial step in surgical preparation [7].

Despite the clear evidence of the impact of improper hand decontamination on the development of healthcare-associated infections, compliance with hand hygiene practice is still low globally. The reasons for non-compliance range from lack of knowledge and inadequate training to non-strict guidelines and a busy working schedule [8].

The current audit revealed that overall compliance was 82% in the first cycle, slightly higher than the compliance observed in other developing countries. For comparison, 63% compliance was observed in an audit conducted at Lahore General Hospital in Pakistan and 71% in an audit conducted in Nepal [9].

Another audit conducted in Sudan, but in a different state (Al-Managil State), also showed lower initial compliance at 64.41%, which improved to 93% after the intervention. However, in our study, there was no improvement in overall compliance in the second cycle despite variations in the individual domains. A compliance rate of 96% was achieved in the third cycle.

The interventions that led to the change in the third cycle included personal demonstrations, instructions, and alerting participants to the posters that had been previously placed in the theatre. Therefore, our study revealed that personal instructions achieved a better outcome than video demonstrations. This was highlighted by two previous studies by Salemi et al. [10] and Mukherjee et al. [11] in India, but contradicts the findings of Weber et al. [12], who concluded that video-based instruction is superior to conventional demonstrations in teaching hand disinfection principles.

According to WHO guidelines, removing hand accessories and ensuring that fingernails are clean and short are crucial steps in hand hygiene. The importance of these steps stems from the fact that both hand accessories and long fingernails can harbor microorganisms that are difficult to remove using standard handwashing techniques. Additionally, hand accessories can lead to physical injury in patients and healthcare professionals and increase the risk of glove perforation and microbial transmission.

In the first cycle, the only category that showed 100% compliance from the outset ensured that the fingernails were clean and short, as observed in studies from Turkey and Nepal, which also demonstrated 100% compliance in maintaining clean nails and removing accessories. In the present study, the latter score was 93%. The high scores in these two categories are attributed to the most visible element of the process.

The lowest compliance was in the duration of scrubbing, as less than one-third (30%) washed their hands for at least two minutes (the shortest recommended time for surgical scrubbing by the WHO). This is a low figure compared to the 100% compliance in an observational study conducted in Nepal. This significant difference could stem from the fact that the guidelines were displayed on computer screen savers in the theatre in Nepal hospital. From this observation, poor practice is primarily influenced by a lack of knowledge and inadequate reinforcement of guidelines.

After handwashing and rinsing, drying hands with towels lowers the risk of SSI because dry hands have less potential to spread bacteria than wet hands [13]. This item had the second-lowest score (70%). Other domains that showed low compliance were holding hands above the elbows during rinsing and drying (73%) and rubbing hands palm to palm (77%) [14].

In the third cycle, an overall compliance rate of 96% was achieved, with 100% compliance in eight out of 15 domains. An improvement of 166% was noted in the hand scrubbing duration item, which increased from 30% to 80% compliance. This again highlights the importance of familiarity with the best practice guidelines [15].

Out of all the steps, the area with the least progress was the proper rinsing of hands and forearms under running water, specifically ensuring the water flowed in one direction from the fingertips to the elbow. This aspect showed minimal improvement compared to others, with only 3.4% correctly rubbing the left wrist with the right hand and 6.7% scrubbing up to the elbow and vice versa.

A notable limitation of this study is its narrow scope, as it was restricted to a single secondary hospital. This limited study area may constrain the generalization of the findings. In addition to potential bias from participants' awareness of being studied, direct observation may have influenced their behavior. This may explain the higher compliance rate in the first cycle compared to other studies conducted in Sudan and other developing countries.

Larger studies involving multiple hospitals of different levels and geographical locations are needed to obtain a better reflection of hand scrubbing practices and the effectiveness of auditing. Another method that is of good value in maintaining long-term compliance, particularly in hospitals with high staff turnover, is a video auditing system, where observation is done via video cameras placed in the operating rooms, which has shown promising results.

The most significant progress was observed in the two-minute scrubbing metric, which increased from 30% to 80%, representing a 166% improvement.

This audit did not include microbiological validation, such as pre- and post-scrub hand swab cultures, to quantify bacterial load reduction. Although the observations were conducted by two doctors, the surgical staff were not informed that they were being individually assessed, which helped minimize observer bias and reduce the Hawthorne effect. Additionally, the audit was conducted in a single referral hospital, which may limit the generalizability of the findings.

Continuous education and training on surgical hand hygiene are essential to sustain compliance. Incorporating modules on the link between hand hygiene and SSIs, as well as emphasizing the potential financial savings from reduced postoperative infections, could strengthen institutional infection control strategies.

## Conclusions

The audit uncovered notable deficiencies in hand hygiene practices among surgical residents and house officers at Prince Osman Digna Referral Hospital. Initial adherence to the standard and appropriate guidance was moderate. Critically, only a minimum percentage of participants met the recommended minimum scrubbing time of two minutes, highlighting a significant area for improvement.

Following targeted interventions, including live demonstrations, video tutorials, and strategically placed instructional posters, compliance rates improved substantially. By the third audit cycle, overall adherence had reached a high improvement. The most significant progress was observed in the two-minute scrubbing metric. Participant feedback indicated a strong preference for hands-on demonstrations, finding them more effective than video-based instruction. This audit underscores the value of structured educational initiatives and direct feedback in promoting adherence to infection control protocols. Ongoing training and regular re-evaluation will be vital to sustaining these gains. Ultimately, these findings underscore the crucial importance of proper hand hygiene in reducing SSIs and enhancing patient outcomes.
